# Supplementary material for: Terpyridine-metal complexes: effects of different substituents on their physico-chemical properties and density functional theory studies
Source: R Soc Open Sci. 2020 Nov 25;7(11):201208. doi: 10.1098/rsos.201208 (PMC7735333; doi:10.1098/rsos.201208)
Supplement: Revised SI (15-09-2020)-RSOS.docx [file rsos201208supp1.docx]

Supporting information file for manuscript

**Terpyridine-metal complexes: Effects of different substituents on their physico-chemical properties and DFT studies**

Ehsan Ullah Mughal^[[1]](#footnote-1)^*^a^, Masoud Mirzaei^[[2]](#footnote-2)^*^b^, Amina Sadiq^c^, Sana Fatima^a^, Ayesha Naseem^a^, Nafeesa Naeem^a^, Nighat Fatima^d^, Samia Kausar^a^, Ataf Ali Altaf^a,e^ Muhammad Naveed Zafar^f^, Bilal Ahmad Khan^g^

*^a^Department of Chemistry, University of Gujrat, Gujrat-50700, Pakistan*

*^b^Department of Chemistry, Faculty of Science, Ferdowsi University of Mashhad, Mashhad, Iran*

*^c^Department of Chemistry, Govt. College Women University, Sialkot-51300, Pakistan*

*^d^Department of Pharmacy, COMSATS University Islamabad, Abbottabad Campus-22060, Pakistan*

*^e^Department of Chemistry, University of Okara, Okara-56300, Pakistan*

*^f^Department of Chemistry, Quaid-i-Azam University, Islamabad-45320, Pakistan*

*^g^Department of Chemistry, University of Azad Jammu and Kashmir, Muzaffarabad, Pakistan*

| ***UV-Vis Spectra of Some of the Synthesized Compounds (Table-S1)*** | ***P1*** |
| --- | --- |
| ***Emission Spectra of Some of the Synthesized Compounds (Table-S2)*** | ***P8*** |
| ***Anti-Microbial Activities of Some of the Synthesized Compounds (Table-S3)*** | ***P18*** |
| ***Representative FTIR Spectra of Some of the Synthesized Compounds (Figure-S1)*** | ***P24*** |
| ***Comparison of FTIR Data of the Synthesized Compounds*** | ***P27*** |
| ***Representative NMR Spectra of the Synthesized Ligands (Figure-S2)*** | ***P28*** |
| ***Representative MALDI-ToF Spectra of Some of the Synthesized Compounds (Figure-S3)*** | ***P36*** |
| ***Representative FMO Studies of Some of the Synthesized Compounds (Figure-S4)*** | ***P38*** |

**Table S1. UV-Vis Spectra of Some of the Synthesized Compounds**

| **Compound No.** | **UV Spectra** |
| --- | --- |
| **L_3_** | 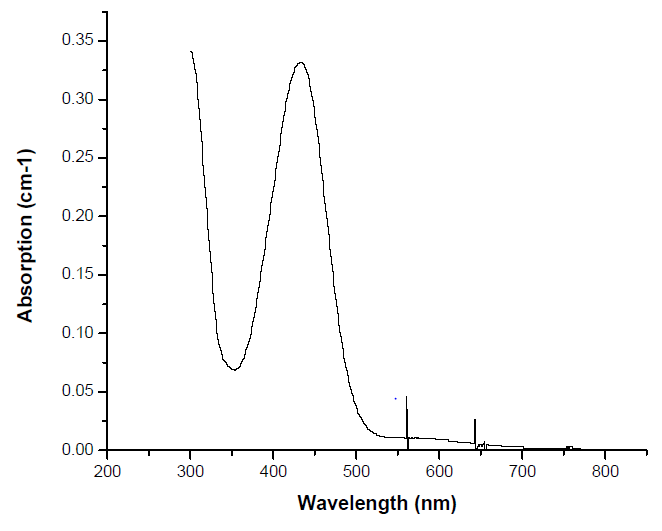 |
| **L_8_** |  |
| **C_1_** |  |
| **C_2_** |  |
| **C_3_** |  |
| **C_4_** |  |
| **C_5_** |  |
| **C_6_** |  |
| **C_7_** |  |
| **C_8_** |  |
| **C_9_** |  |
| **C_10_** |  |
| **C_11_** |  |
| **C_12_** |  |
| **C_13_** |  |
| **C_14_** |  |
| **C_15_** |  |
| **C_16_** |  |
| **C_17_** |  |
| **C_18_** |  |
| **C_19_** |  |
| **C_20_** |  |
| **C_21_** |  |
| **C_22_** |  |
| **C_23_** |  |
| **C_24_** |  |
| **C_25_** |  |
| **C_26_** |  |
| **C_27_** |  |

**Table S2. Emission Spectra of Some of the Synthesized Compounds**

| **Compound No.** | **Emission Spectra** |
| --- | --- |
| **L_8_** |  |
| **L_8_ + C_22_, C_23_, C_24_** |  |
| **C_1_** |  |
| **C_2_** |  |
| **C_3_** |  |
| **C_4_** |  |
| **C_5_** |  |
| **C_6_** |  |
| **C_7_** |  |
| **C_8_** |  |
| **C_9_** |  |
| **C_10_** |  |
| **C_11_** |  |
| **C_12_** |  |
| **C_13_** |  |
| **C_14_** |  |
| **C_15_** |  |
| **C_16_** |  |
| **C_17_** |  |
| **C_18_** |  |
| **C_19_** |  |
| **C_20_** |  |
| **C_21_** |  |
| **C_22_** |  |
| **C_23_** |  |
| **C_24_** |  |
| **C_25_** |  |
| **C_26_** |  |
| **C_27_** |  |

**Table S3. Anti-Microbial Activities of Some of the Synthesized Compounds**

| **Compound No.** | **Bacterial /Fungal**  **Strains** | **Anti-Microbial activities of Some Synthesized Compounds (Well Diffusion Method)** |
| --- | --- | --- |
| **Standard** | ***S. aureus*** | 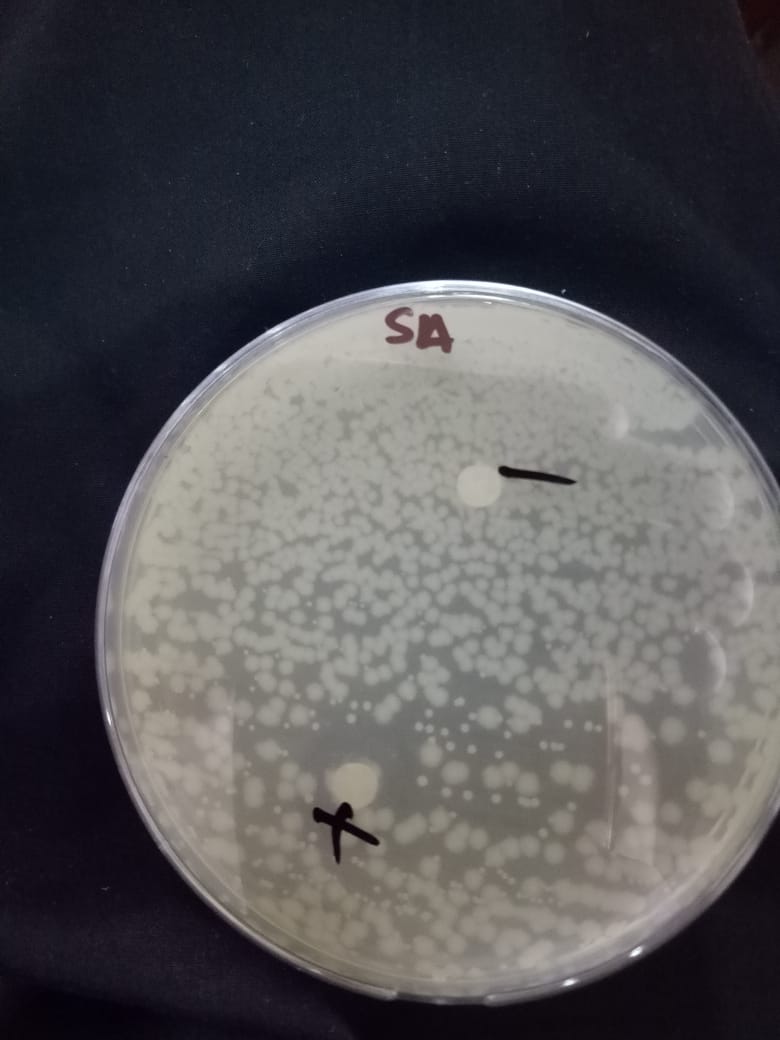 |
| **1 (L_1_)**  **2 (L_2_)**  **3 (L_3_)** | ***S. aureus*** | 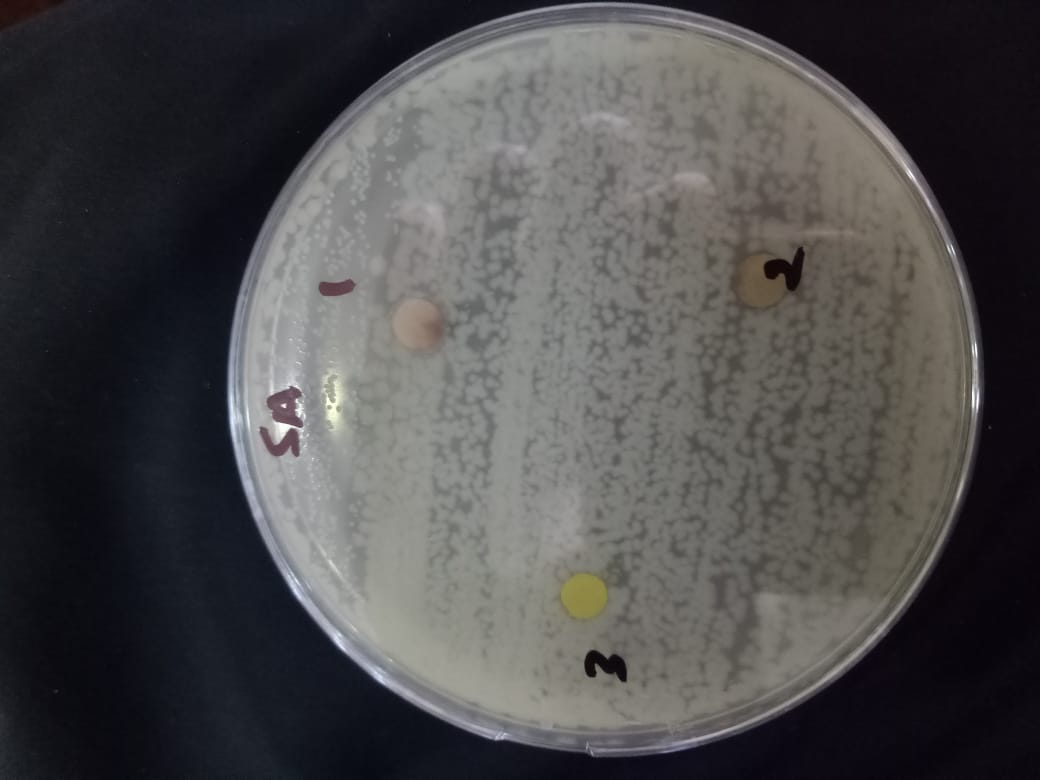 |
| **4 (L_4_)**  **5 (L_5_)**  **6 (L_6_)** | ***S. aureus*** | 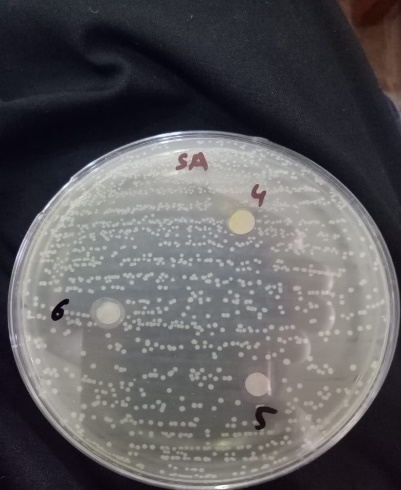 |
| **10 (L_7_)**  **11 (L_8_)**  **12 (L_9_)** | ***S. aureus*** | 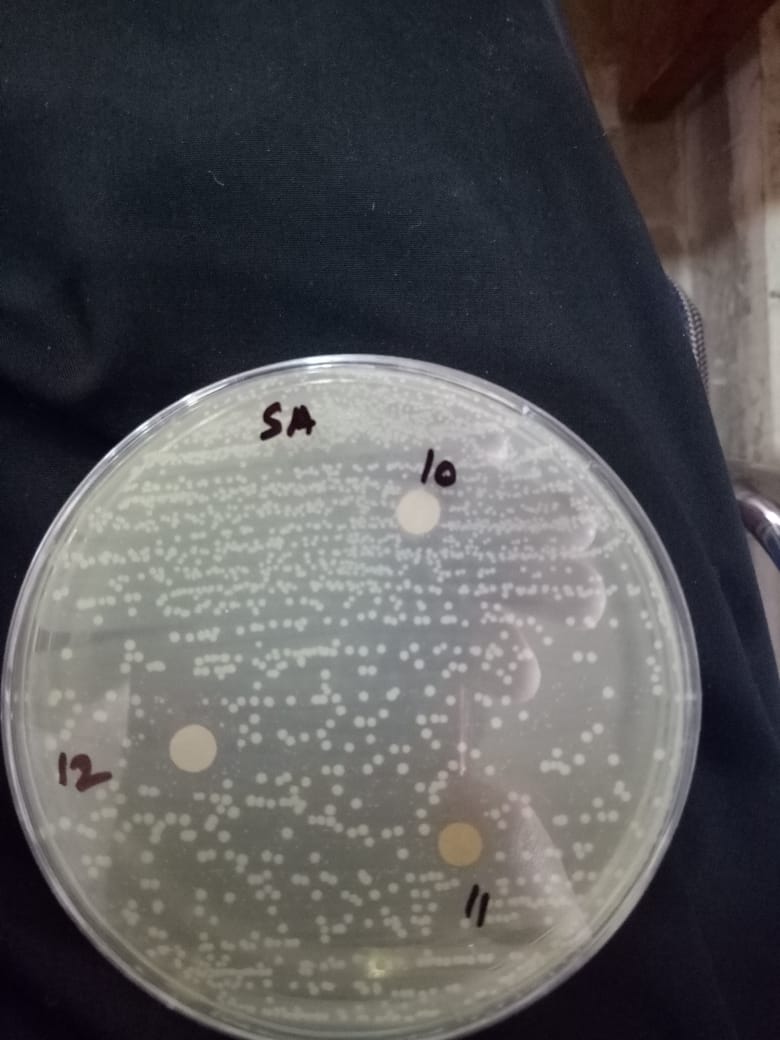 |
| **15 (C_1_)** | ***S. aureus*** | 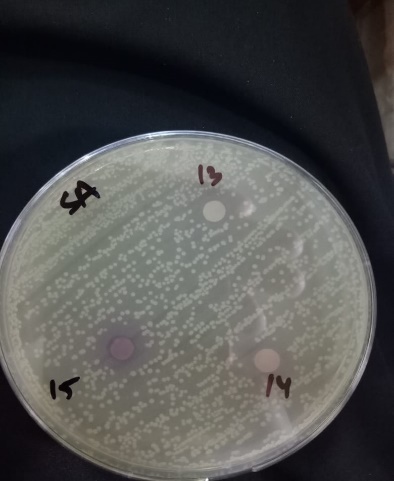 |
| **16 (C_3_)**  **17 (C_6_)**  **18 (C_9_)** | ***S. aureus*** | 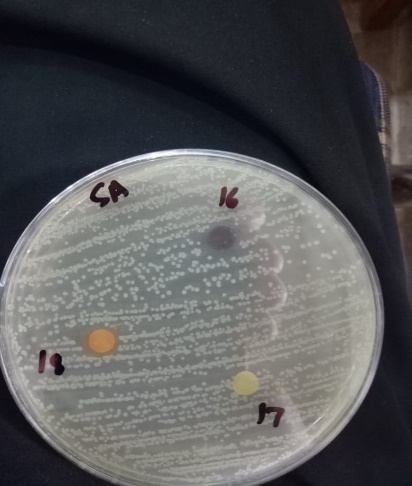 |
| **19 (C_22_)**  **20 (C_23_)**  **21 (C_24_)** | ***S. aureus*** | 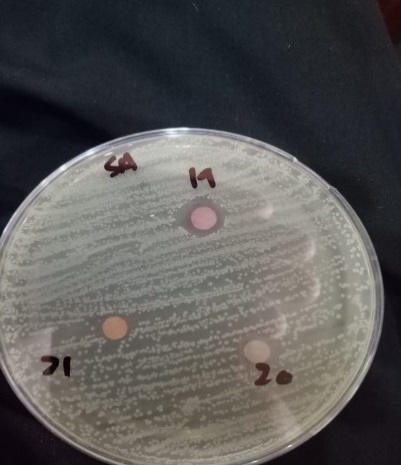 |
| **Standard** | ***E.coli*** | 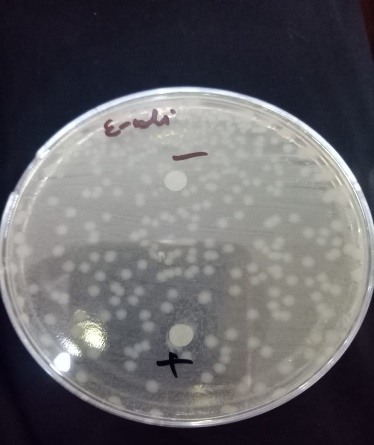 |
| **1 (L_1_)**    **2 (L_2_)**  **3 (L_3_)** | ***E.coli*** | 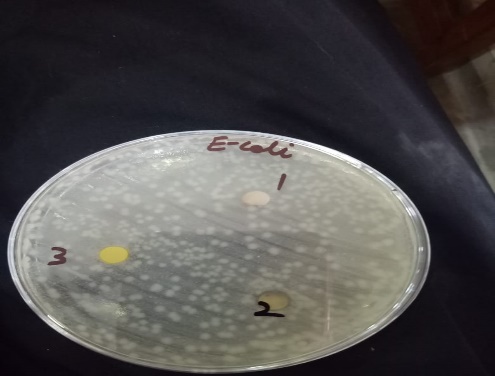 |
| **4 (L_4_)**  **5 (L_5_)**  **6 (L_6_)** | ***E.coli*** | 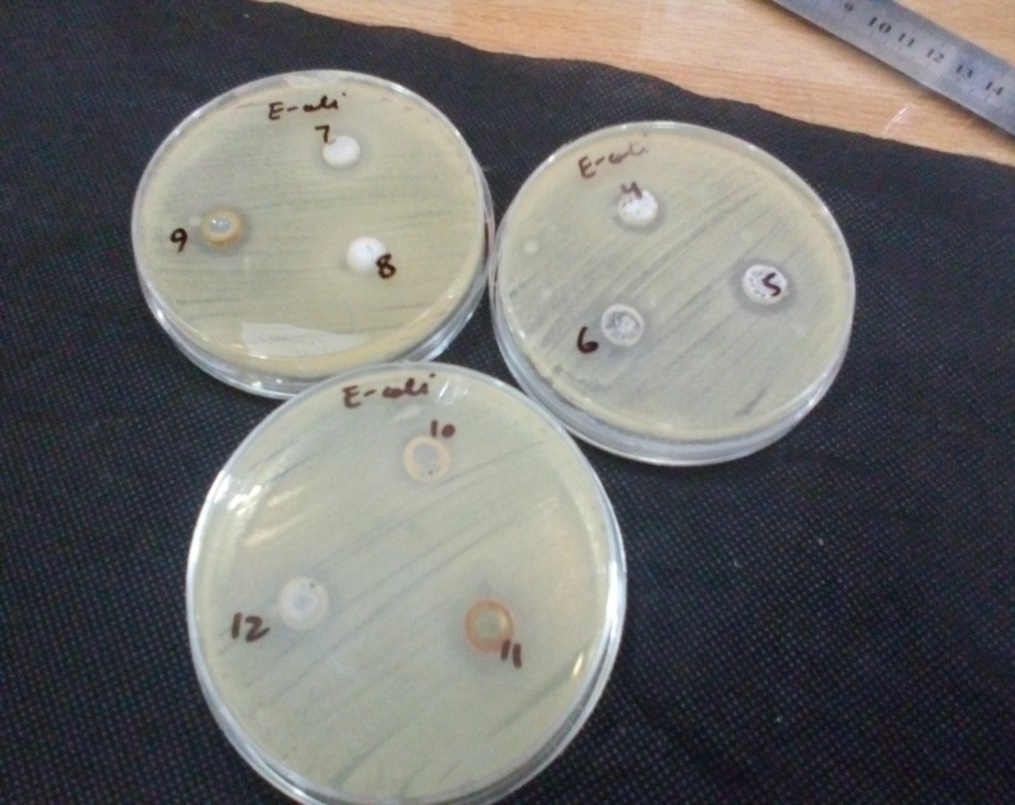 |
| **10 (L_7_)**  **11 (L_8_)**  **12 (L_9_)** | ***E.coli*** | 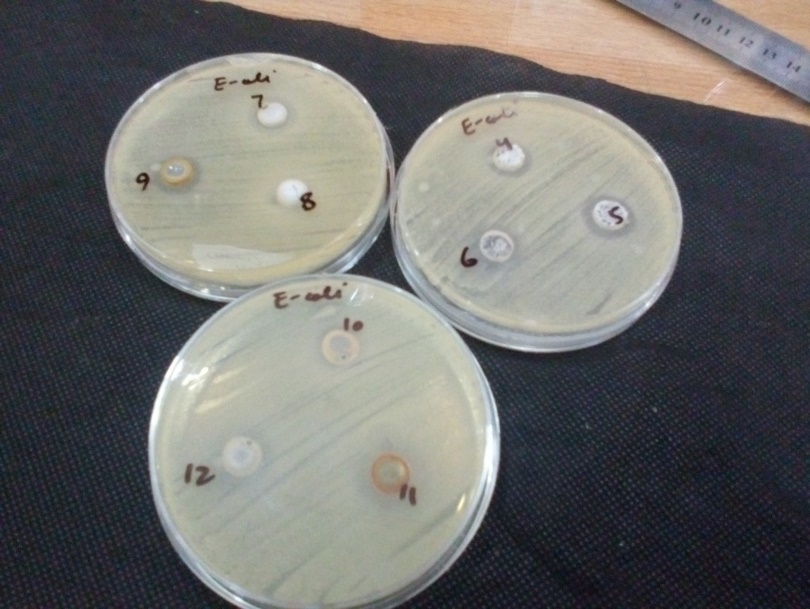 |
| **15 (C_1_)** | ***E.coli*** | 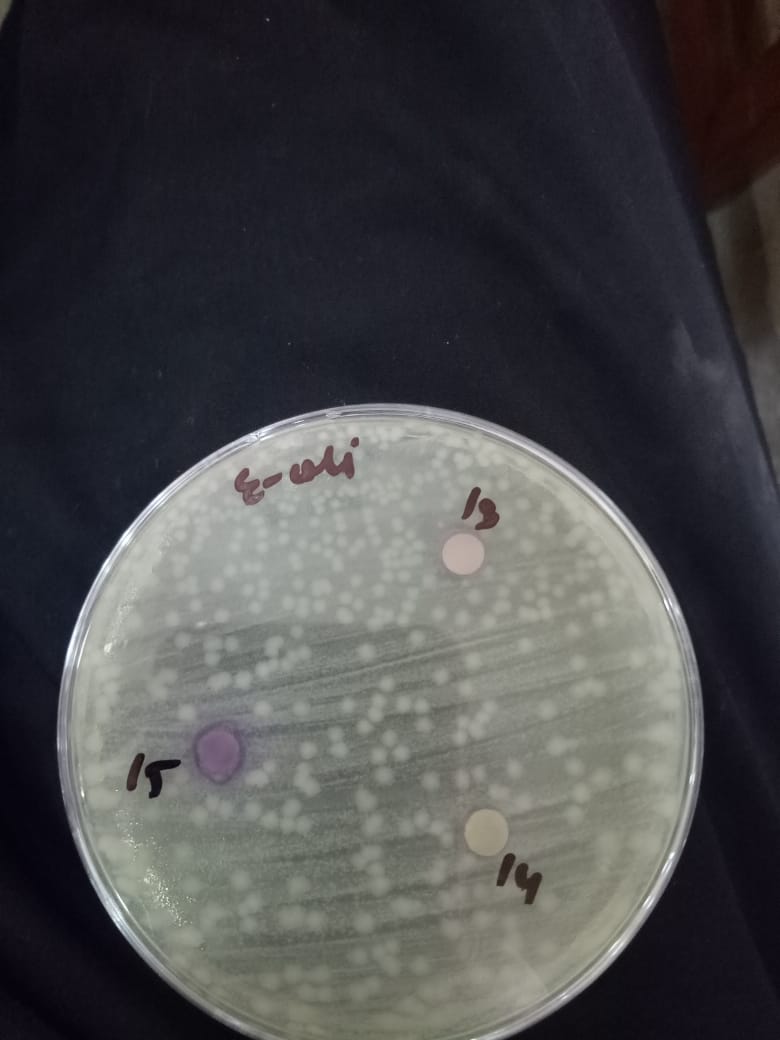 |
| **16 (C_3_)**  **17 (C_6_)**  **18 (C_9_)** | ***E.coli*** | 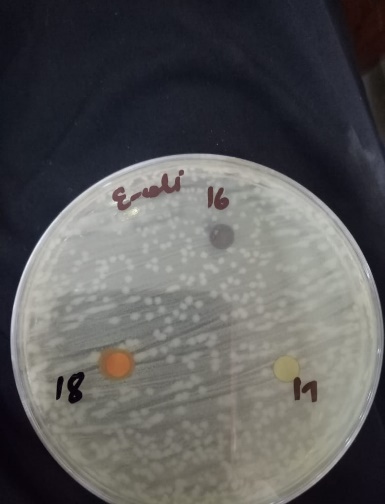 |
| **19 (C_22_)**  **20 (C_23_)**  **21 (C_24_)** | ***E.coli*** | 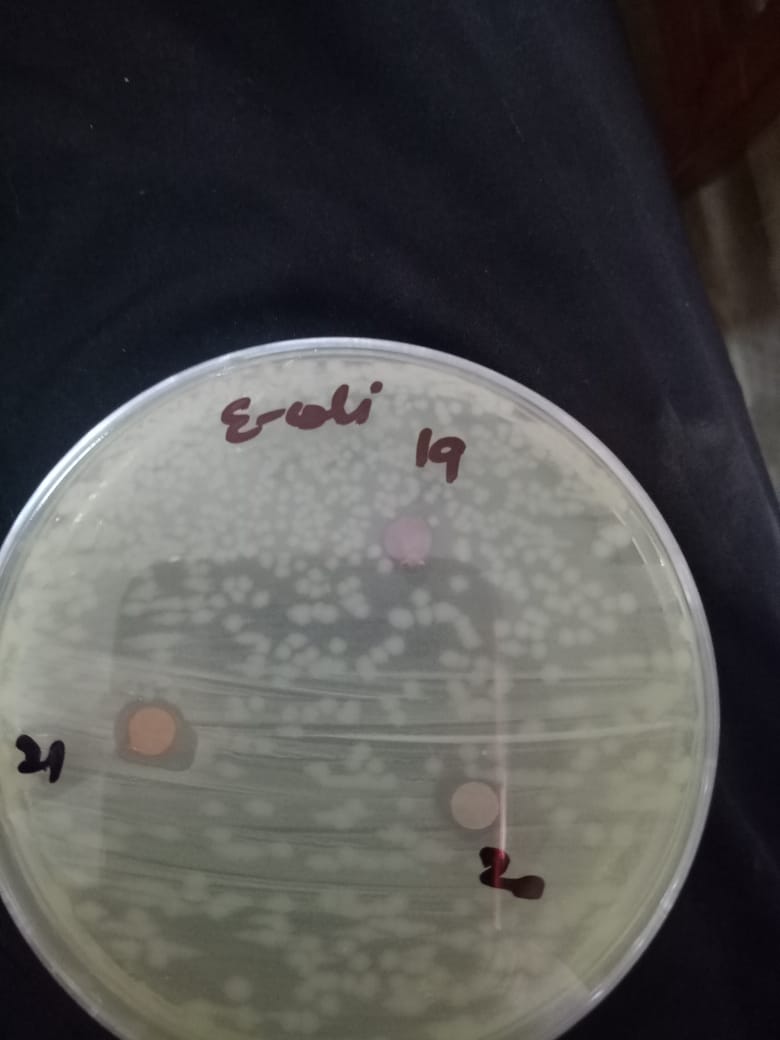 |
| **1 (L_1_)**  **2 (L_2_)**  **3 (L_3_)** | ***P. aeruginosa*** | 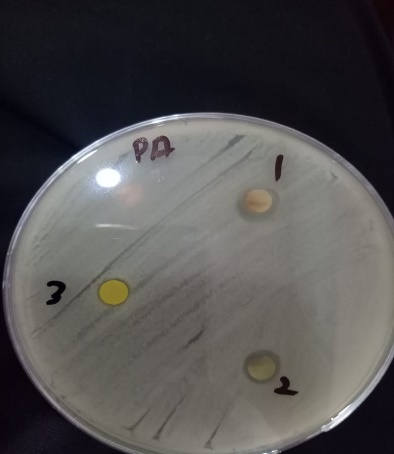 |
| **4 (L_4_)**  **5 (L_5_)**  **6 (L_6_)** | ***P. aeruginosa*** | 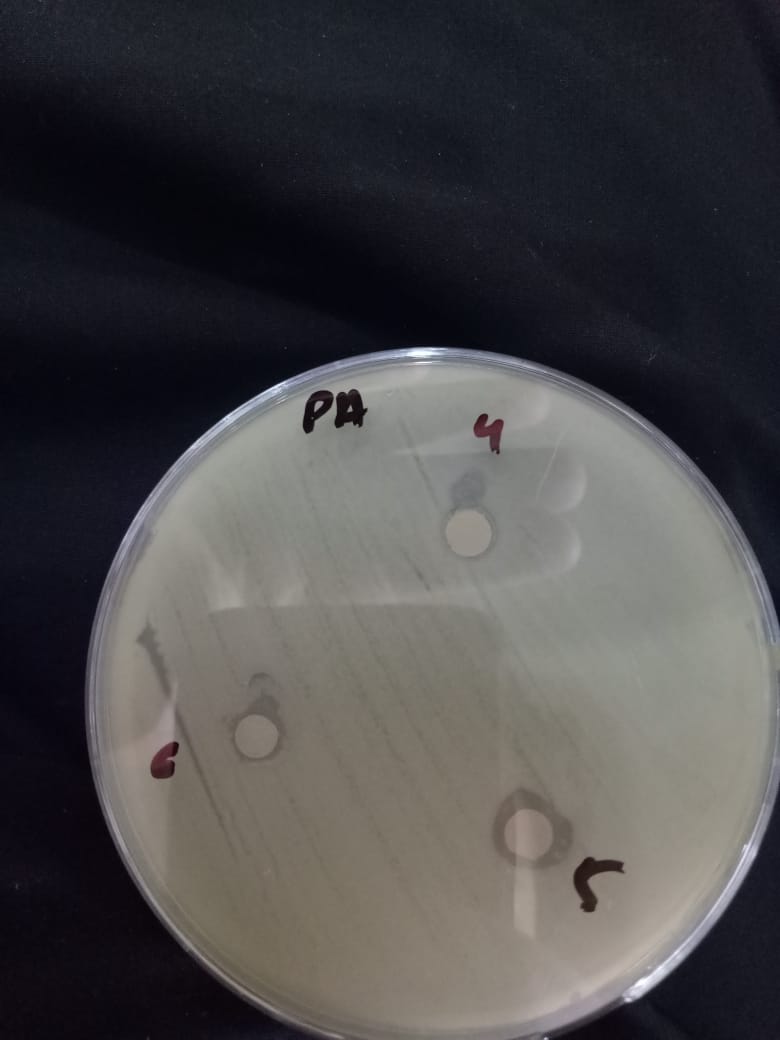 |
| **10 (L_7_)**  **11 (L_8_)**  **12 (L_9_)** | ***P. aeruginosa*** | 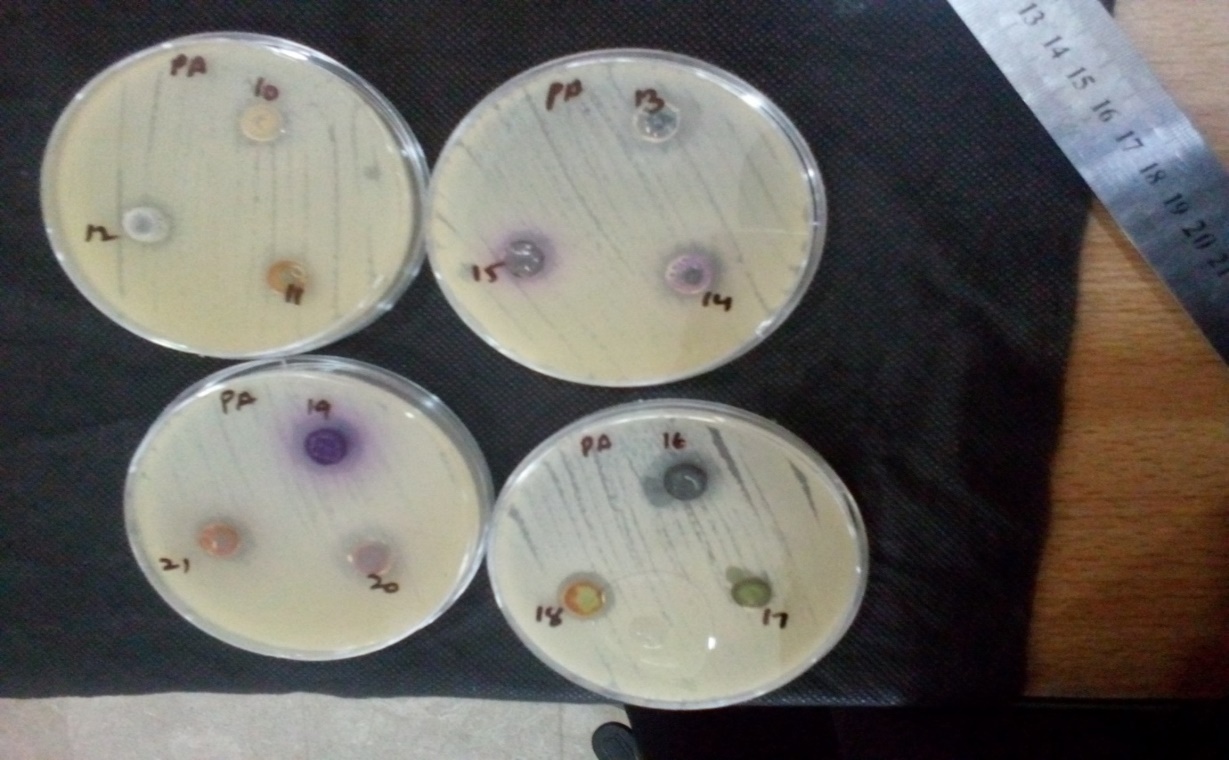 |
| **15 (C_1_)** | ***P. aeruginosa*** | 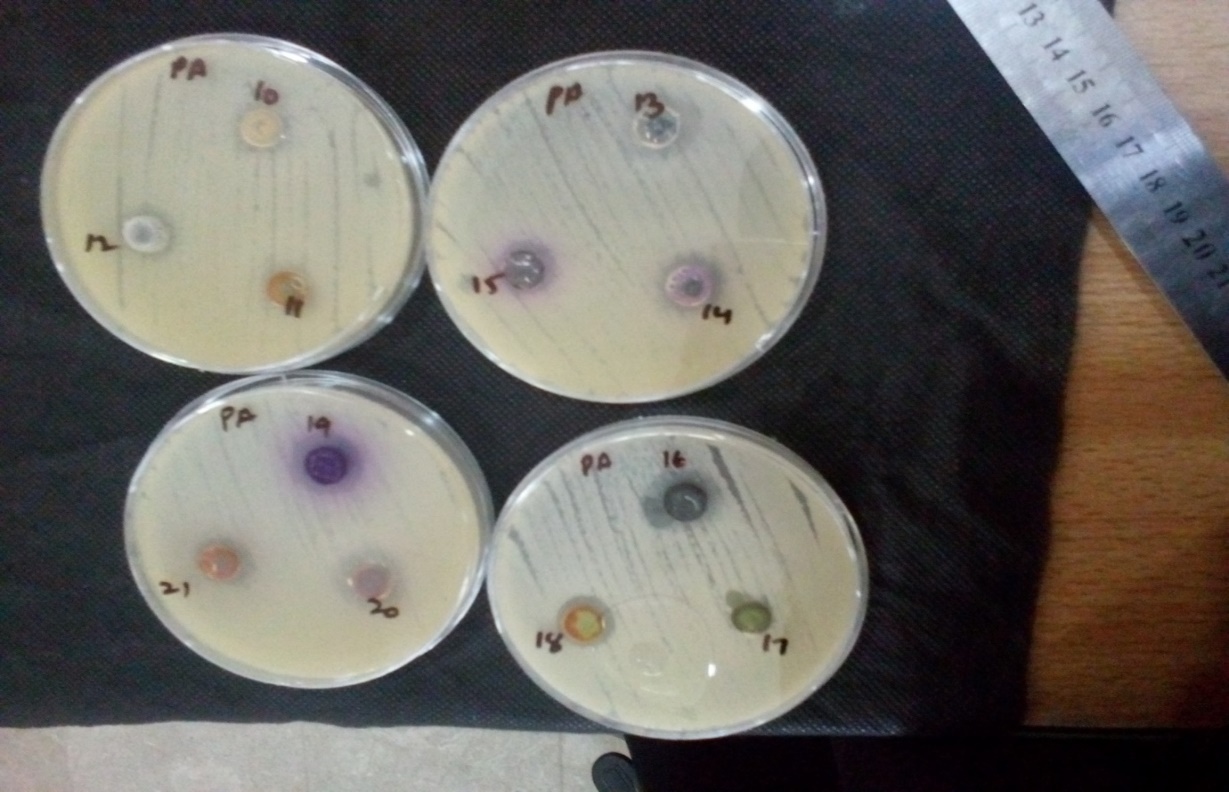 |
| **16 (C_3_)**  **17 (C_6_)**  **18 (C_9_)** | ***P. aeruginosa*** | 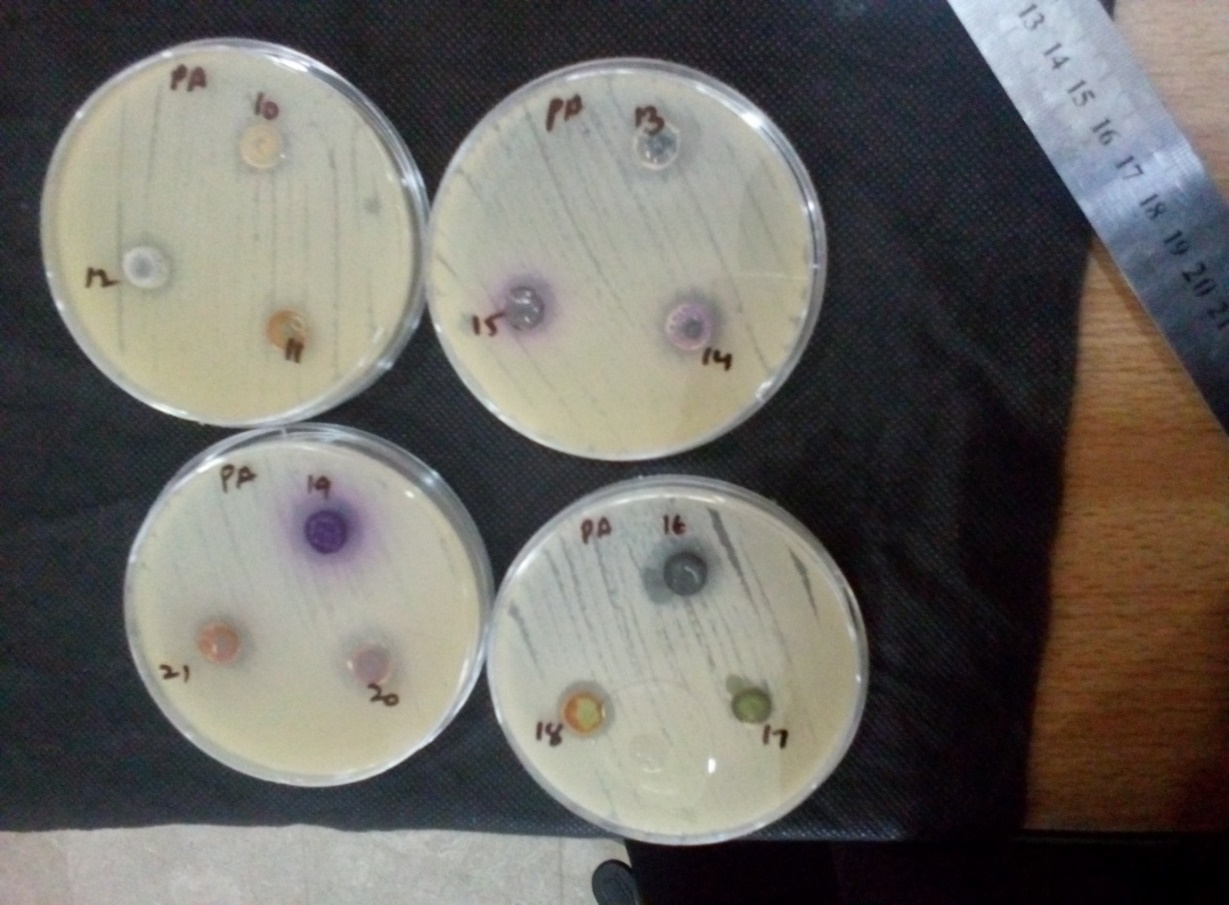 |
| **19 (C_22_)**  **20 (C_23_)**  **21 (C_24_)** | ***P. aeruginosa*** | 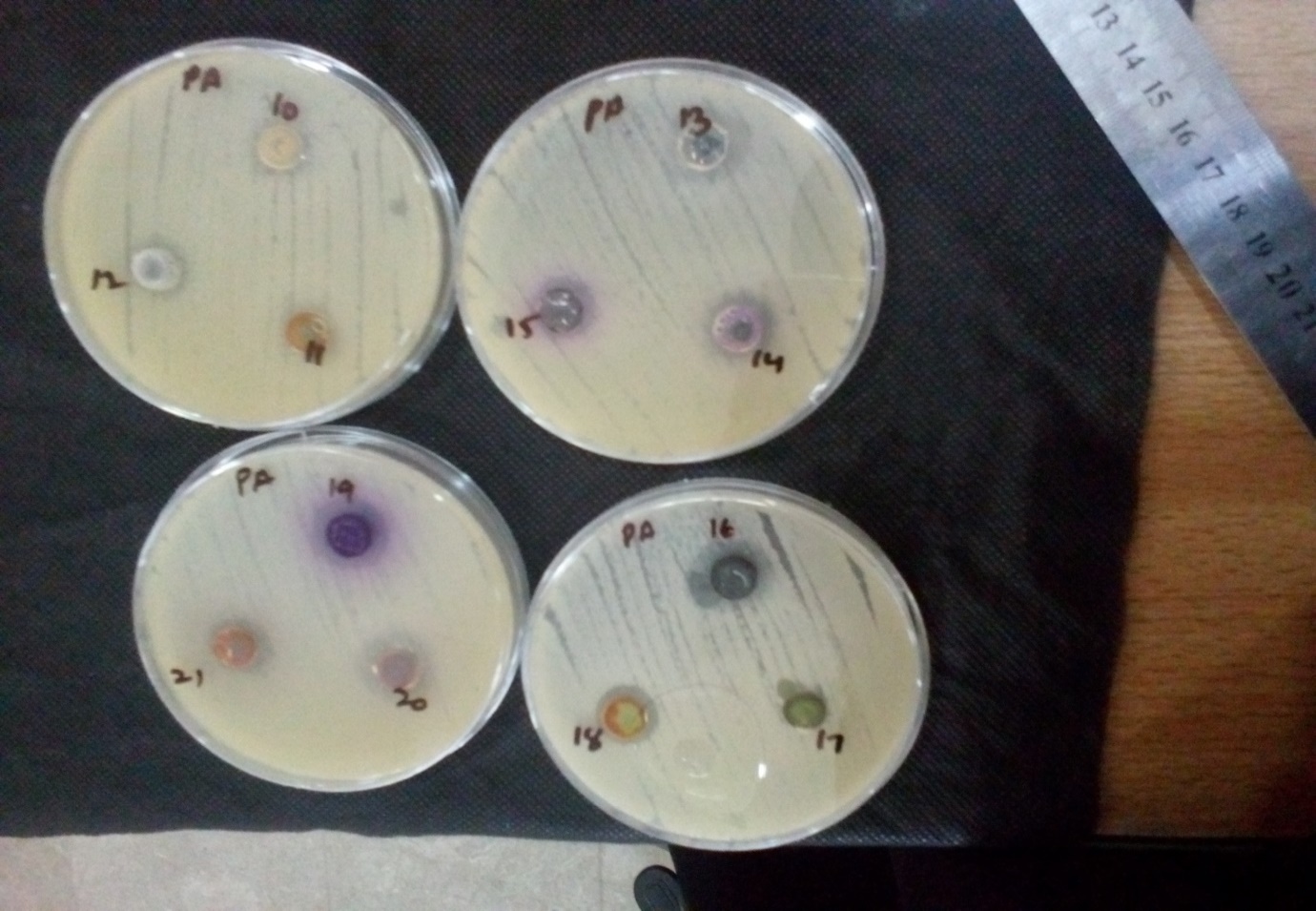 |
| **Standard** | ***C. albicans*** | 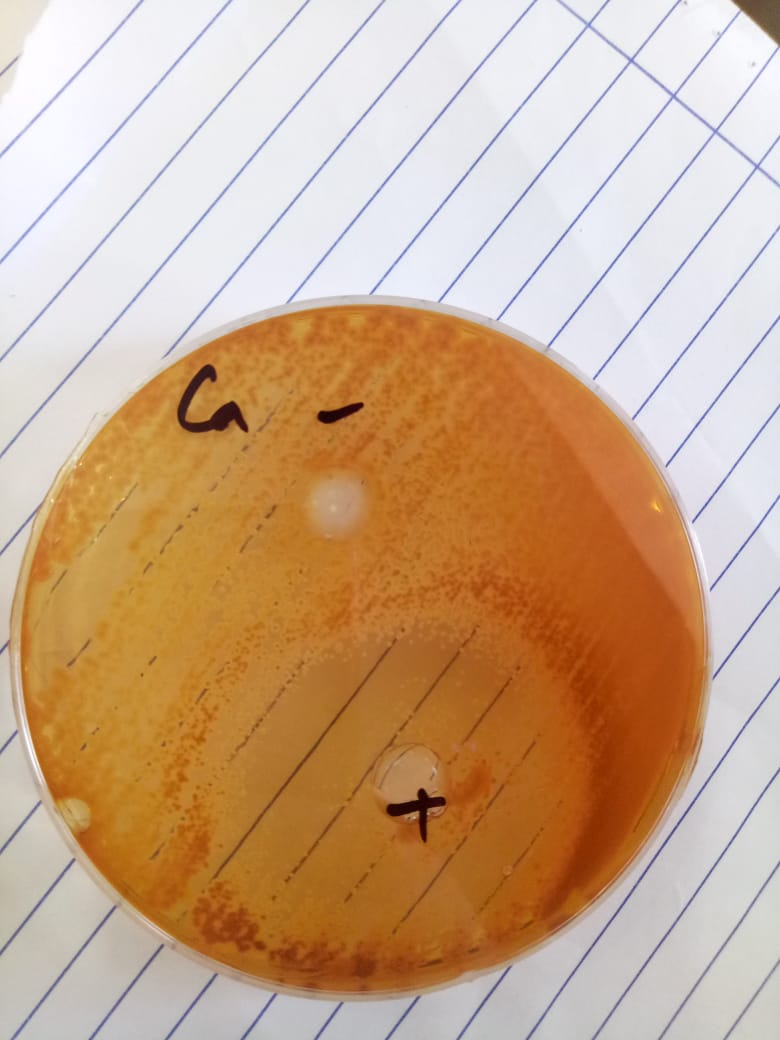 |
| **16 (C_3_)**  **17 (C_6_)**  **18 (C_9_)** | ***C. albicans*** | 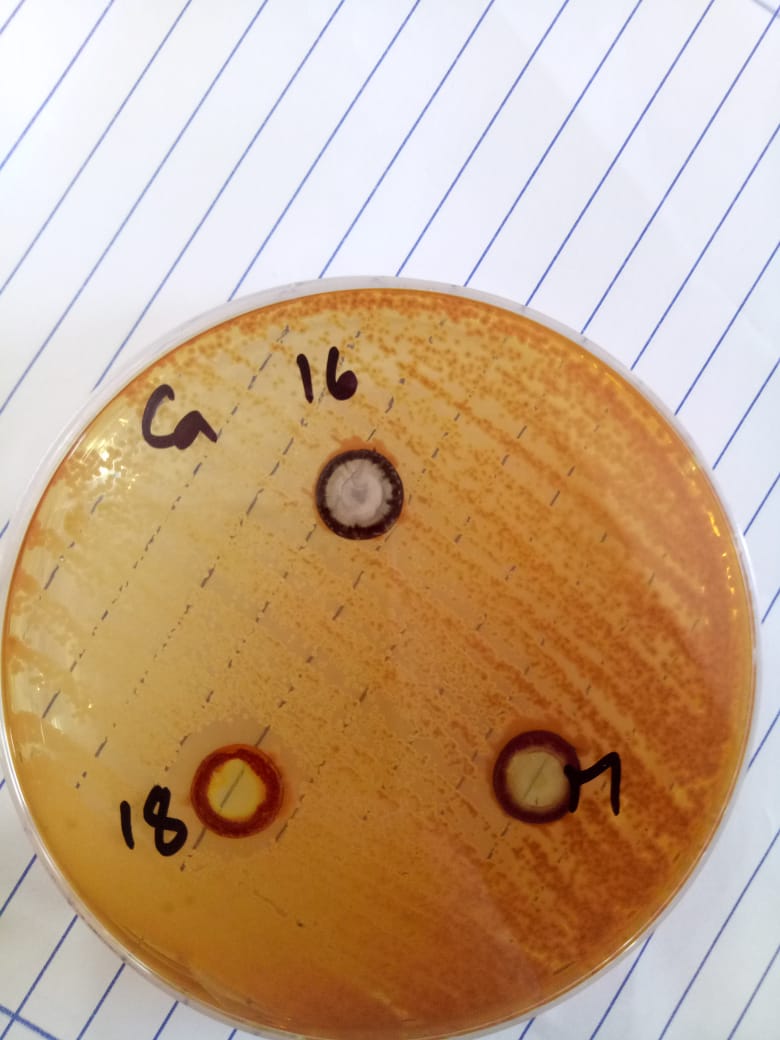 |
| **19 (C_22_)**  **20 (C_23_)**  **21 (C_24_)** | ***C. albicans*** | 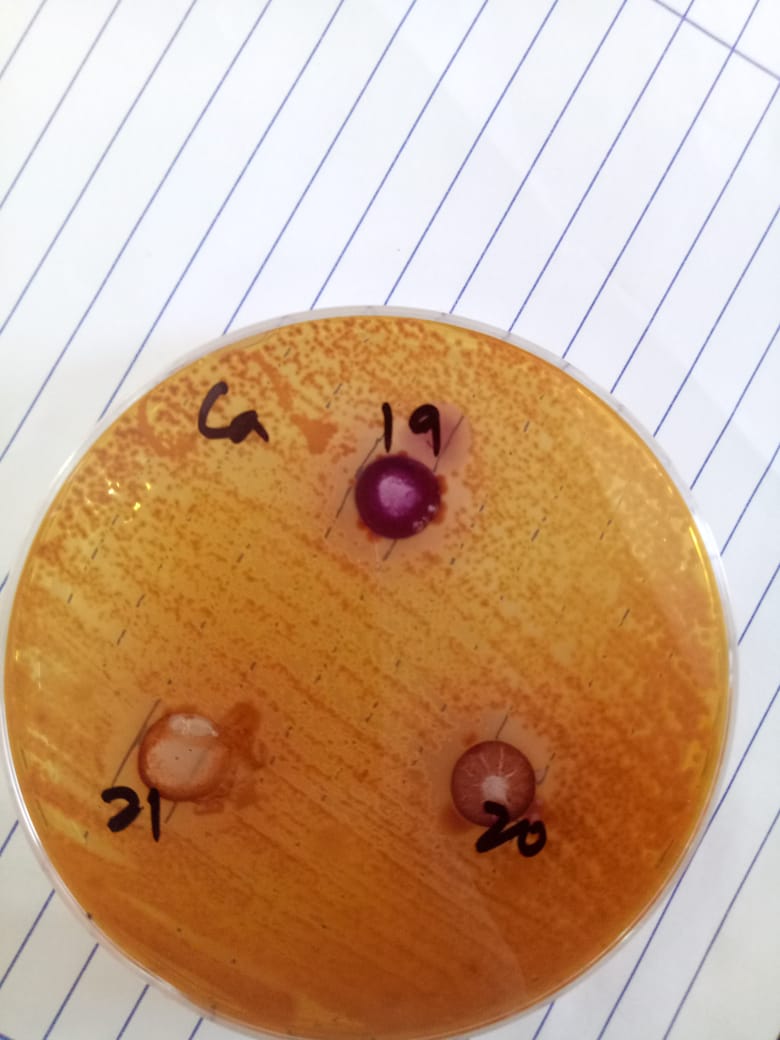 |

**Figure S1. Representative FTIR Spectra of Some of the Synthesized Compounds**

**FTIR Spectrum of (L_8_)**

**FTIR Spectrum of (C_22_)**

**FTIR Spectrum of (C_24_)**

**Comparison of IR spectral band shift of complexes with respective ligands**

The coordination of substituted terpyridine ligands with Zn(II), Co(II) and Fe(II) were confirmed by comparing IR spectra of the free ligands with those of the complexes. The chelation of the 2,2ʹ-6ʹ,2ʹʹ-terpyridine ligands can be easily detected by monitoring the position of the ligand bands which have shifted to lower or high energy upon complexation. Absorption due to C-H can be observed in the IR spectra of all the ligands at around 3375 - 3000 cm^-1^ due to methine group in the terpyridine ligands. This indicates that only one of the C-H atoms from each ligand is deprotonated and involved in coordination with Zn(II), Co(II) and Fe(II). In addition, the band appeared in the region 1307–1270 cm^-1^ due to C–N stretching in the free ligands has been downward shifted to 1268-1230 cm^-1^ in the IR spectra of the complexes indicating the coordination through nitrogen atom. In all complexes, the ν (C=N) underwent a change in frequency and intensity, caused by complexation and upshifted by 20-60 cm^-1^ to higher frequency. The coordination of azomethine nitrogen to Zn(II), Co(II) and Fe(II) are further corroborated with the appearance of a new band at around 578 - 630 cm^-1^ region due to a ν (Zn–N), ν (Co–N), and ν (Fe–N), stretch in the spectra of all complexes.

**Figure S2: NMR Spectra of the Synthesized Ligands**

**Note:** Due to compatibility issue between the data and word-file, there are some tilted lines on the NMR spectra. We apologize for that and request to ignore those errors, please

**^1^H-NMR Spectrum of L_3_**

**^13^C-NMR Spectrum of L_3_**

**^1^H-NMR Spectrum of L_5_**

**^13^C-NMR Spectrum of L_5_**

**^1^H-NMR Spectrum of L_7_**

**^13^C-NMR Spectrum of L_7_**

**^1^H-NMR Spectrum of L_8_**

**^13^C-NMR Spectrum of L_8_**

**Figure S3: Representative MALDI-ToF Mass Spectra of Some of the Synthesized Compounds**

**MALDI-ToF Mass Spectra of Complex (C_8_)**

1. **Full Spectrum (MS = 1011.3310)**


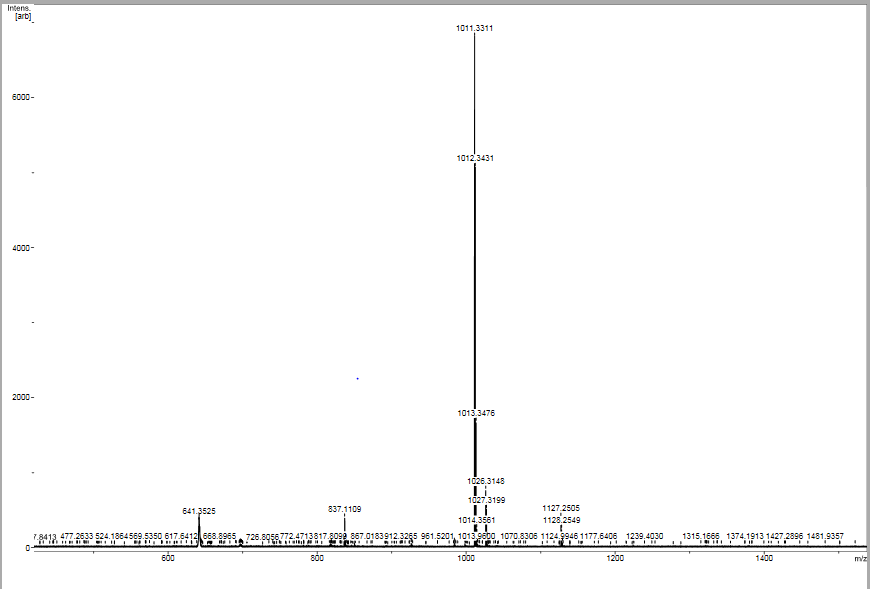


1. **With High Resolution**


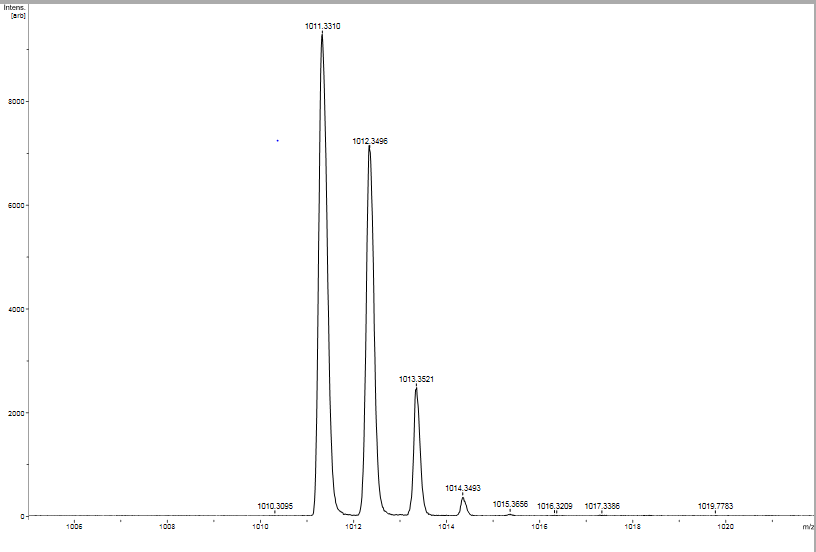


**MALDI-ToF Mass Spectra of Complex (C_24_)**

1. **Full Spectrum (MS = 764.1584)**


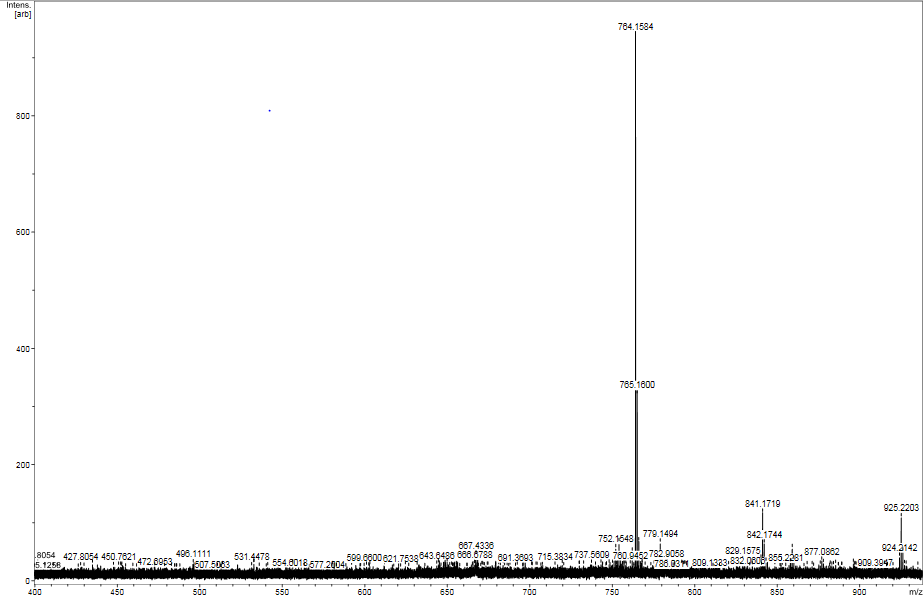


1. **With High Resolution**


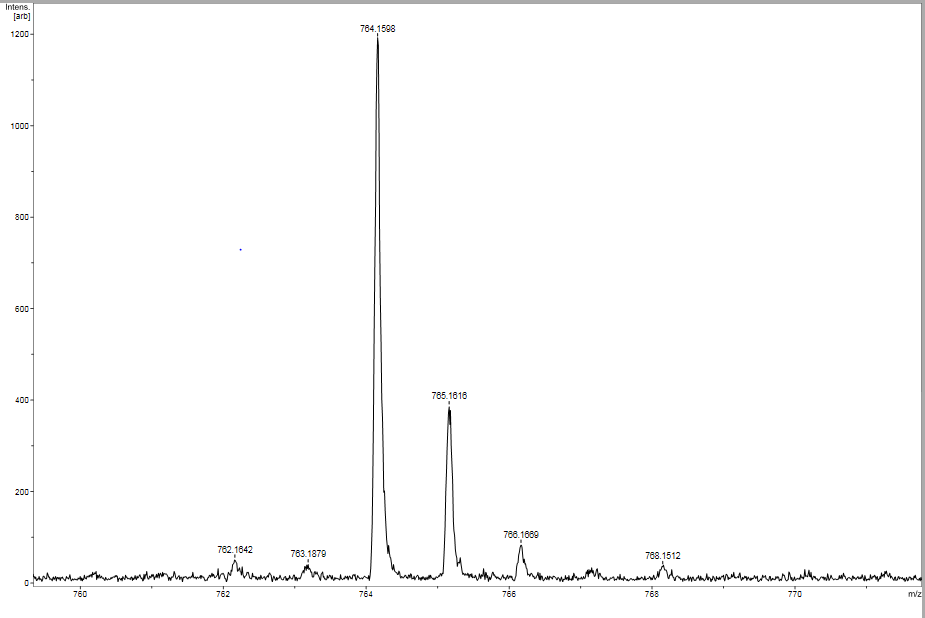


**Figure S4: Representative FMO Studies of Some of the Synthesized Compounds**

**DFT-B3LYP^*^ (DZ) calculated Frontier Molecular Orbitals (FMOs) of C_13_**


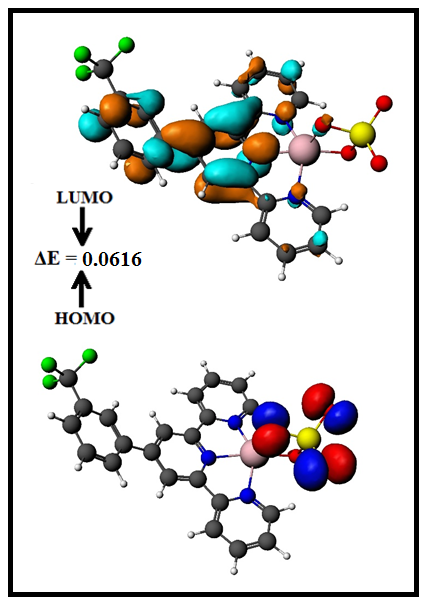


**DFT-B3LYP^*^ (DZ) calculated Frontier Molecular Orbitals (FMOs) of C_14_**


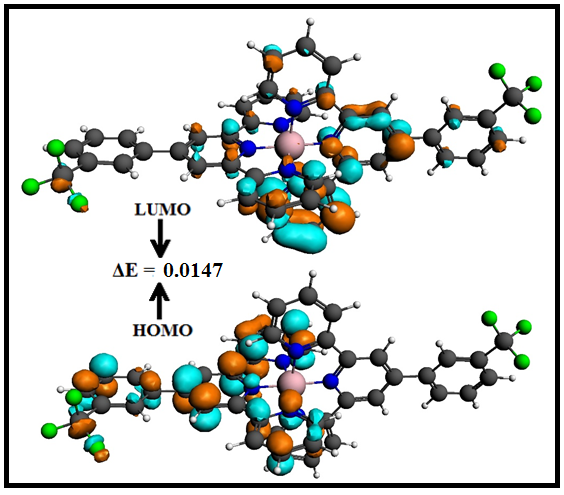


**DFT-B3LYP^*^ (DZ) calculated Frontier Molecular Orbitals (FMOs) of C_22_**


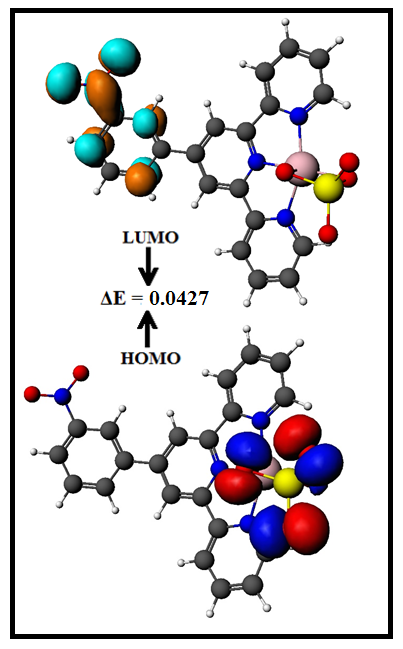


**DFT-B3LYP^*^ (DZ) calculated Frontier Molecular Orbitals (FMOs) of C_23_**


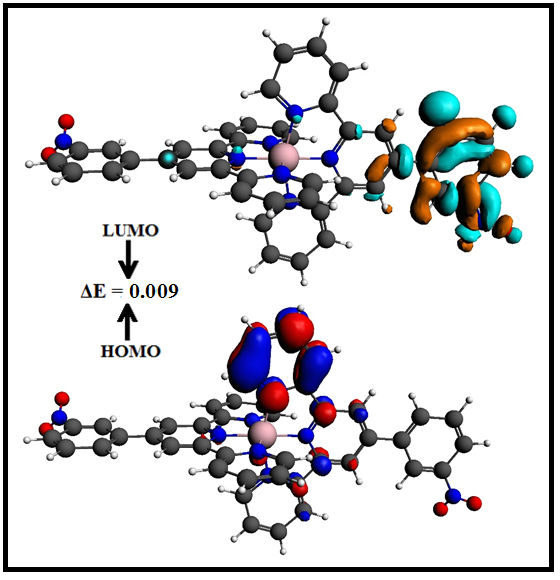


1. *Corresponding Authors: [ehsan.ullah@uog.edu.pk](mailto:*ehsan.ullah@uog.edu.pk); Tel.: +92-333-8464959 [↑](#footnote-ref-1)
2. *Corresponding Authors: [mirzaeesh@um.ac.ir](mailto:mirzaeesh@um.ac.ir); Fax.: +98-051-38796416 [↑](#footnote-ref-2)
